# Design of an international multicentre RCT on group schema therapy for borderline personality disorder

**DOI:** 10.1186/s12888-014-0319-3

**Published:** 2014-11-18

**Authors:** Pim Wetzelaer, Joan Farrell, Silvia MAA Evers, Gitta A Jacob, Christopher W Lee, Odette Brand, Gerard van Breukelen, Eva Fassbinder, Heather Fretwell, R Patrick Harper, Anna Lavender, George Lockwood, Ioannis A Malogiannis, Ulrich Schweiger, Helen Startup, Teresa Stevenson, Gerhard Zarbock, Arnoud Arntz

**Affiliations:** Department of Clinical Psychological Science, Faculty of Psychology and Neuroscience, Maastricht University, Universiteitssingel 40, 6229 ER Maastricht, P.O. Box 616, 6200 MD Maastricht, The Netherlands; Department of Psychology, Indiana University-Purdue University Indianapolis, Administrative Office, 402 N Blackford, LD 124, Indianapolis, IN 46202 USA; Center for Borderline Personality Disorder Treatment & Research, Indianapolis, USA; Department of Health Services Research, CAPHRI School of Public Health and Primary Care, Faculty of Health Medicine and Life Sciences, Maastricht University, Duboisdomein 30, 6229 GT Maastricht, P.O. Box 616, 6200 MD Maastricht, The Netherlands; Trimbos Institute, The Netherlands Institute of Mental Health and Addiction, Utrecht, The Netherlands; Department of Clinical Psychology and Psychotherapy, Institute for Psychology, University of Freiburg, Engelbergerstrasse 41, 79085 Freiburg, Germany; Department of Psychology and Exercise Science, Murdoch University, 90 South St, Murdoch, WA 6153 Australia; De Viersprong, The Netherlands Institute for Personality Disorders, De Beeklaan 2, Postbus 7, 4661 EP Halsteren, The Netherlands; Department of Methodology and Statistics, Faculty of Health Medicine and Life Sciences, Maastricht University, Peter Debyeplein 1, P.O. Box 616, 6200 MD Maastricht, The Netherlands; Faculty of Psychology and Neuroscience, Maastricht University, Universiteitssingel 40, P.O. Box 616, 6200 MD Maastricht, The Netherlands; Department of Psychiatry and Psychotherapy, University of Lübeck, Ratzeburger Allee 160, 23538 Lübeck, Germany; Midtown Mental Health/ Eskenazi Health, 5610 Crawfordsville Rd Suite 22, Indianapolis, IN 46224 USA; Department of Psychiatry, Indiana University School of Medicine, Indianapolis, USA; Bradford District Care Trust, Bradford, UK; South London and Maudsley NHS Foundation Trust, London, UK; Schema Therapy Institute Midwest, 471 West South Street, Suite 41C, Kalamazoo, MI 49007 USA; 1st Department of Psychiatry, Eginition Hospital, Medical School, Athens University, 72-74, Vas. Sofias Ave, 115 28 Athens, Greece; Greek Society of Schema Therapy, 17, Sisini str, 115 28 Athens, Greece; Klinik für Psychiatrie und Psychotherapie, University of Lübeck, Ratzeburger Allee 160, 23538 Lübeck, Germany; Peel and Rockingham Kwinana Mental Health Service, Cnr Clifton and Ameer Street, Rockingham, P.O. Box 288, WA 6968 Australia; IVAH GmbH (Institute for Training in CBT), Hans-Henny-Jahnn-Weg 51, 22085 Hamburg, Germany; Department of Clinical Psychology, University of Amsterdam, Weesperplein 4, 1018 XA Amsterdam, The Netherlands

**Keywords:** Borderline personality disorder, Group schema therapy, RCT, Economic evaluation, Cost-effectiveness

## Abstract

**Background:**

Borderline personality disorder (BPD) is a severe and highly prevalent mental disorder. Schema therapy (ST) has been found effective in the treatment of BPD and is commonly delivered through an individual format. A group format (group schema therapy, GST) has also been developed. GST has been found to speed up and amplify the treatment effects found for individual ST. Delivery in a group format may lead to improved cost-effectiveness. An important question is how GST compares to treatment as usual (TAU) and what format for delivery of schema therapy (format A; intensive group therapy only, or format B; a combination of group and individual therapy) produces the best outcomes.

**Methods/Design:**

An international, multicentre randomized controlled trial (RCT) will be conducted with a minimum of fourteen participating centres. Each centre will recruit multiple cohorts of at least sixteen patients. GST formats as well as the orders in which they are delivered to successive cohorts will be balanced. Within countries that contribute an uneven number of sites, the orders of GST formats will be balanced within a difference of one. The RCT is designed to include a minimum of 448 patients with BPD. The primary clinical outcome measure will be BPD severity. Secondary clinical outcome measures will include measures of BPD and general psychiatric symptoms, schemas and schema modes, social functioning and quality of life. Furthermore, an economic evaluation that consists of cost-effectiveness and cost-utility analyses will be performed using a societal perspective. Lastly, additional investigations will be carried out that include an assessment of the integrity of GST, a qualitative study on patients’ and therapists’ experiences with GST, and studies on variables that might influence the effectiveness of GST.

**Discussion:**

This trial will compare GST to TAU for patients with BPD as well as two different formats for the delivery of GST. By combining an evaluation of clinical effectiveness, an economic evaluation and additional investigations, it will contribute to an evidence-based understanding of which treatment should be offered to patients with BPD from clinical, economic, and stakeholders’ perspectives.

**Trial registration:**

Netherlands Trial Register NTR2392. Registered 25 June 2010.

**Electronic supplementary material:**

The online version of this article (doi:10.1186/s12888-014-0319-3) contains supplementary material, which is available to authorized users.

## Background

Borderline personality disorder (BPD) is a common mental disorder characterised by enduring and pervasive patterns of instability in interpersonal relationships, identity, impulsivity, and affect [1]. The prevalence of BPD in the general population, as revealed by recent community surveys that use DSM-IIIR or DSM-IV criteria, is estimated at 0.5 to 2.7% (median =0.7%) [2]. BPD prevents patients from developing their full potential and leading a fulfilling life. Consequently, many patients do not finish their education, or complete it at a suboptimal level. Similarly, many have a job beneath their capacity, or they have no job at all. BPD patients often engage in problematic relationships, self-injury, suicide attempts, and substance abuse. Furthermore, 8-10% of BPD patients end their lives prematurely due to suicide [3].

BPD severely impairs quality of life across mental, social and physical dimensions [4]. In a Swedish study that compared quality of life between women with BPD and a normal population, it was found that women with BPD were significantly impaired in all domains, including physical, emotional, cognitive and sexual functioning [5]. Relationships with their family and partner were also found to be impaired [5]. Two Dutch studies have shown that the burden of disease for both adolescent and adult patients with various personality disorders, including BPD, is severe and their quality of life is markedly impaired [6,7].

In addition to the devastating effects of BPD on the functioning of patients, it imposes a large burden on their families, friends, and society as a whole. Families and friends may face the challenging task of providing informal care [8,9], whereas society bears the costs of a more intensive use of health services [6,10-13], productivity losses [10,13], and other inter-sectorial costs [14]. In clinical settings, BPD patients are regarded as notoriously difficult to treat, leading many therapists to refrain from treating them. In the absence of robust evidence for the effectiveness of any specific medication for BPD [15], psychotherapy is, currently, the most promising strategy for its treatment.

Schema therapy (ST) is delivered as an outpatient treatment with the intention of bringing about full recovery. It has proven more clinically effective than transference-focused psychotherapy (TFP) in a randomized controlled trial (RCT) comparing both treatments head-to-head [16]. Results from the same RCT also show that it has a high probability of being more cost-effective than TFP [17]. It was found that ST could be transported out of the clinical trial to use in the regular Dutch healthcare setting with no loss of clinical effectiveness [18]. In these studies, all treatments led to an improvement in quality of life above and beyond recovery from symptoms.

ST can also be delivered in a group format, thus enabling a more efficient use of resources. In addition, initial findings have indicated that this format can increase the effectiveness of ST [19]. Psychotherapy groups can provide a family-like environment to patients, giving them a sense of belonging and facilitating the secure attachments needed for limited reparenting (a defining element of ST that refers to the therapist trying, within the bounds of a professional relationship, to meet a patient’s unfulfilled core emotional needs). Furthermore, patients can accept the responses of peers as more ‘genuine’ than those of the therapist, whose responses are, at least initially, often viewed as less ‘real’ and more professional. An RCT on group schema therapy (GST) has demonstrated its effectiveness for the treatment of BPD [19]. Treatment time in this study was relatively short (eight months in contrast to up to three years for studies on individual ST). This suggests that GST leads to faster recovery than individual ST. However, this seminal investigation still leaves important questions unanswered. First, since this RCT was performed by the developers of GST it is unknown how effective GST is when delivered by other therapists in other centres. Second, in this RCT GST was an addition to treatment as usual (TAU) for patients who were already receiving TAU beforehand. GST has thus not been tested as an integral and stand-alone treatment. Third, this RCT was not accompanied by an economic evaluation, and hence, does not give insight into the cost-effectiveness of GST. Fourth, in this RCT TAU was very ineffective. Because TAU has improved in recent years, due to the dissemination of evidence-based treatments and recent insights from studies, GST needs to be compared to up-to-date TAU.

A second study on GST for BPD was a Dutch pilot study in which two cohorts of BPD patients (total number of 18 patients) were treated with the combination of group and individual ST [20]. This study showed large effect sizes on a broad range of outcomes including improvements in BPD symptomatology, general psychopathological symptoms and quality of life. However, this study was uncontrolled and also did not assess cost-effectiveness [20]. In sum, findings on the clinical effectiveness of GST from previous studies are promising yet leave open important questions that need to be answered before the further dissemination of GST is supported.

To answer these questions an international, multicentre RCT on GST for BPD will be performed. This article provides a description of the study design. The main study objective is to compare the clinical effectiveness and cost-effectiveness of GST and TAU. The RCT involves two formats of GST, one that consists almost exclusively of GST and one that combines GST with individual ST. Both formats have a two year duration. Group and individual schema therapy are, to a large extent, considered complementary and mutually supportive. Individual sessions may have an advantage over group sessions in that the therapist is in a better position to motivate a patient for treatment, to offer extended trauma processing, and to offer a more in depth attachment. In contrast, the group sessions may provide important connection experiences that deal with fundamental issues of BPD. For example, a stronger sense of connection provided by the group may do more to counter abandonment fears and sharing common experiences among peers might add to a decrease in a patient’s sense of isolation and/or defectiveness. On the one hand, combining group and individual schema therapy may offer potentially synergistic effects [20]. On the other hand, the availability of individual sessions might lead to some patients avoiding full participation in the group, thus reducing its potential curative power. Hence, no specific hypothesis about the superiority of either format has been formulated. To evaluate the relative contribution of the proposed formats to outcome, a secondary objective is to compare the two formats of GST. This will help to establish the optimal format for delivery of GST to patients with BPD.

Furthermore, a series of additional substudies will be performed. These consist of an assessment of the integrity of GST, a qualitative investigation into the experiences of patients and therapists with GST and an investigation of variables that might influence the change process of GST and thereby affect outcomes such as dropout rates and patient improvement. Qualitative data will be collected from patient and therapist interviews and/or focus groups regarding their experiences of specific aspects of GST. This will provide information on which aspects of the GST protocol are most beneficial and any aspects that may be less helpful or problematic. This will not only help in identifying how the different components of ST can affect outcome, but also in deciding which format is preferred. Based on this information, GST can then be further tailored to the needs of the primary stakeholders before its further dissemination.

## Methods/Design

In this RCT an evaluation of clinical effectiveness, a full economic evaluation and a series of additional investigations will be performed. Primarily, GST (format A or B) will be compared to TAU and, secondarily, GST formats A and B will be compared.

### Design

**Figure 1:**
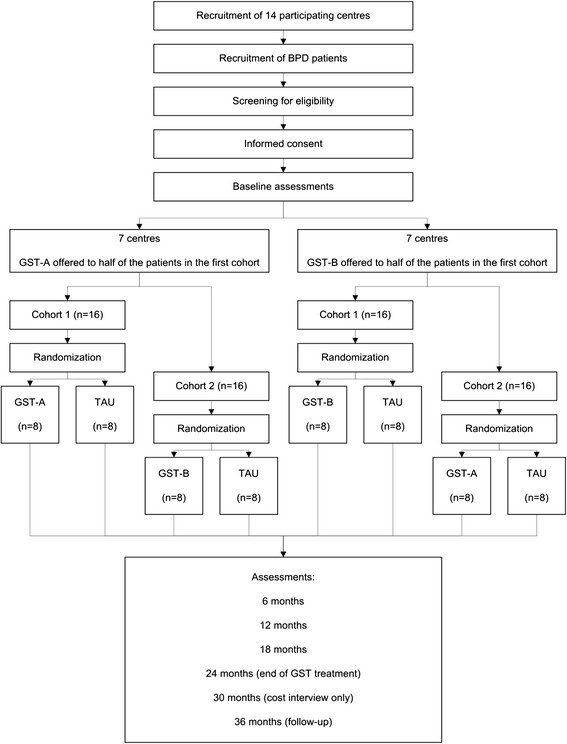
**Flow chart of the study design.** Patients with BPD are recruited at 14 participating centres and screened for eligibility. After informed consent is signed, baseline assessments are performed. Subsequently, patients are randomized in blocks of two per centre to either GST or TAU. Half of the centres offer GST-A to the first cohort of patients and the other half offers GST-B to the first cohort of patients. In two Dutch sites, a third cohort is recruited which is randomly assigned to either format for GST so that the total number of cohorts receiving both formats is balanced. Assessments are performed approximately every six months for the first two years, after which GST treatment ends. Costs are also assessed at approximately 30 months. Follow-up assessments take place 36 months after randomization.

A multicentre RCT will be conducted with participating centres (at the time of this writing) in the following countries: six centres in the Netherlands, three in Germany, one in Australia, two in the UK, one in the USA and one in Greece. Some centres that initially agreed to participate had to withdraw because of budget cuts resulting from economic difficulties that made participation impossible. One Dutch centre withdrew due to recruitment rates being too slow and was replaced by another centre. In contrast, two Dutch centres’ expeditious recruitment rates permitted the inclusion of a third cohort. This can compensate for additional centres that agreed to participate but may still withdraw, or for centres that fail to recruit the planned minimum of 32 patients per centre. Patient flow, screening, randomization and assessments are displayed schematically in Figure 1. The research protocol has been approved by the Medical Ethics Committee of Maastricht University for the Dutch sites; by the Murdoch University Human Research Ethics Committee for the Australian sites; by the Ethics Committee of the Albert-Ludwigs-University Freiburg, the Ethics Committee of the University of Lübeck and the Ethics Committee of the Psychotherapist Association Hamburg for the German sites; by the Ethics Committee of the Eginition Hospital, Medical School, University of Athens for the Greek site; and by the National Research Ethics Service Committee London - Camberwell St Giles for the British sites. Ethical review is in process in the USA.

### Recruitment

Patients will be recruited in the participating centres. They will be invited to participate in the screening process when diagnosed with BPD or when this is suspected. Both patients who are already receiving treatment for BPD as well as new referrals can be included as participants. After reading and hearing information on the RCT and signing an informed consent, patients will be assessed for in- and exclusion criteria.

### Patients

Patients are eligible when they (1) are between 18 and 65 years of age, (2) have a primary diagnosis of BPD, (3) have a BPD severity score of above 20 on the Borderline Personality Disorder Severity Index, version IV [21], (4) are willing to participate in the study and (5) are able to participate in (group) treatment and research for 2 years. Patients will be excluded if they have a lifetime psychotic disorder (except for a brief psychotic disorder as described in the Diagnostic and Statistical Manual of mental disorders, version IV (DSM-IV) BPD criterion 9); an IQ below 80; if they are unable to read, speak or write the language used at the centre; if they have Attention Deficit Hyperactivity Disorder (ADHD) (if successfully treated, ADHD is not an exclusion criterion), bipolar disorder type 1, dissociative identity disorder, full or sub threshold narcissistic or antisocial personality disorder (PD), substance dependence needing clinical detoxification, a serious and/or unstable medical illness or if they have received schema therapy of more than three months duration in the last three years. Well-trained clinicians will diagnose patients at baseline using Structured Clinical Interviews for DSM-IV axis I and II disorders (SCID I and II). Screening for ADHD will be performed using a six item version of the World Health Organization (WHO) ADHD screener [22]. If this screener indicates the presence of ADHD, then the patient will be further assessed with the ADHD section of the Structural Clinical Interview for DSM-IV, Childhood Diagnosis (KID-SCID) to examine whether ADHD was present during primary school age to exclude false positives.

### Sample size

Previous findings of Farrell et al. [19] indicate an outcome difference between GST and TAU with an effect size of d around 2 (d refers to Cohen’s d [23]). However, some shrinkage of the effect might be expected when GST is provided by centres that were not involved in the development of GST. Also, although TAU was virtually ineffective in the abovementioned study, recent meta-analyses and RCTs of modern treatments for BPD suggest that these treatments can be effective [24,25]. Hence, the current RCT is designed to have sufficient power to detect an effect size of d = 0.5.

With respect to the comparison between the two formats for GST, no large outcome differences are expected. Whereas small differences are unlikely to influence the preferences of patients and therapists, medium differences may. Therefore, sufficient power is needed to detect an effect size of d = 0.5 between the two formats of GST.

Over the course of three years a dropout of 20% is expected, with about 5% in the first year. Assuming the effects of GST and TAU become apparent after one year [19], the study is powered taking into account a 5% attrition rate. Later attrition is partly compensated by including all randomized patients in analysis. Furthermore, since conservative effect sizes were chosen, the calculated initial sample size could be larger than needed and may therefore also compensate for attrition over 5%.

To test whether GST is superior to TAU and assuming centre as random effect and a centre by treatment interaction that corresponds to a typical intraclass correlation coefficient (ICC) of almost 0.05 in line with literature [26,27], 236 patients (which implies 8 centres of 32 patients, 16 receiving TAU and 16 receiving GST) are needed to detect a difference of d = 0.5 with 90% power using a two-tailed significance level of α = 0.05. For the comparison between GST-A and GST-B and again assuming a typical intraclass correlation of nearly 0.05, a sample size is needed of 202 patients (which implies 13 centres of 16 patients, 8 receiving GST-A and 8 receiving GST-B) to have 90% power to detect a difference of d = 0.5 at a two-tailed significance level of α = 0.05. Taking into account the expected 5% attrition, one additional centre is needed. To be able to balance the orders in which GST formats are delivered to successive cohorts, an even number of sites is required. Including 14 centres gives 90% power to detect a difference of d = 0.40 between GST and TAU and a difference of d = 0.50 between GST-A and GST-B, both at a two-tailed significance level of α = 0.05 (a detailed explanation of the sample size calculation is provided in Additional file 1).

### Randomization

Patients will be randomized centrally in blocks of two per centre (GST versus TAU) using a computer-generated list by an independent central research assistant after baseline screening is complete and all in/exclusion criteria have been checked by this assistant. Each centre will have at least two cohorts of at least sixteen patients of which eight are randomized to GST and eight to TAU. In half of the centres the patients from the first cohort will be randomized to GST format A, which is an intensive group treatment or TAU. The patients from the second cohort will be randomized to GST format B, which combines individual and group treatments or TAU. In the other half of the centres the first cohort will be randomized to GST-B or TAU and the second to GST-A or TAU. The GST formats as well as the orders in which they are delivered to successive cohorts (first GST-A versus TAU, then GST-B versus TAU, and vice versa) will be balanced. Within countries that contribute an uneven number of sites, the numbers of orders of GST formats will have a difference of one.

### Assessments

Prior to randomization, patients will be assessed at baseline. Baseline assessments will be spread over a period of approximately three months on average. Once a cohort is nearly complete, baseline assessments can be speeded up so they are completed within a minimum time period of one month. Inversely, when inclusion is too slow, the maximum time period patients are required to wait before treatment will start is limited to one year. When baseline assessments are complete for all patients, patients will be randomized and treatment will start. Subsequently, patients will be re-assessed approximately every six months over the course of two years. Since the treatment duration for GST is also two years, the fourth assessment after baseline will coincide with GST treatment ending. Follow-up assessments will take place one year later. For the cost interview, a recall period of a year is considered too long. Therefore, an additional assessment will take place midway during the follow-up time period (i.e. two-and-a-half years after start of treatment) during which only the cost interview will be performed.

All assessments will be performed by local research assistants in the centres, except for SCID-interviews which will be done by trained interviewers blind for condition. Assessments include PC-based self-report questionnaires and interviews. An overview of the instruments used per assessment is provided in Table 1.

**Table 1:** **Instruments used per assessment**

	**Screening**	**Baseline** ^*^	**6 months**	**12 months**	**18 months**	**24 months**	**30 months**	**36 months**
SCID I	**•**							**•**
SCID II	**•**							**•**
WHO ADHD screener	**•**							
ITEC	**•**							
BPDSI-IV		**••**	**•**	**•**	**•**	**•**		**•**
GAF		**••**	**•**	**•**	**•**	**•**		**•**
SOFAS		**••**	**•**	**•**	**•**	**•**		**•**
BPD checklist		**•**	**•**	**•**	**•**	**•**		**•**
WSAS		**•**	**•**	**•**	**•**	**•**		**•**
BSI		**•**	**•**	**•**	**•**	**•**		**•**
YSQ-short form		**•**	**•**	**•**	**•**	**•**		**•**
SMI		**•**	**•**	**•**	**•**	**•**		**•**
RSQ		**•**	**•**	**•**	**•**	**•**		**•**
ECNI		**•**	**•**	**•**	**•**	**•**		**•**
EuroQol-5D		**•**	**•**	**•**	**•**	**•**		**•**
WHOQOL-short version		**•**	**•**	**•**	**•**	**•**		**•**
Cost interview		**•**	**•**	**•**	**•**	**•**	**•**	**•**

*Abbreviations*: *SCID I* Structured Clinical Interview for DSM-IV Axis-I Disorders, *SCID-II* Structured Clinical Interview for DSM-IV Axis-II Disorders, *WHO ADHD* World Health Organization Attention Deficit Hyperactivity Disorder, *ITEC* Interview for Traumatic Events in Childhood, *BPDSI-IV* Borderline Personality Disorder Severity Index version IV, *GAF* Global Assessment of Functioning, *SOFAS* Social and Occupational Functioning Assessment Scale, *BPD* Borderline Personality Disorder, *WSAS* Work and Social Adjustment Scale, *BSI* Brief Symptom Inventory, *YSQ* Young Schema Questionnaire, *SMI* Schema Mode Inventory, *RSQ* Relationships Scales Questionnaire, *ECNI* Emotional Core Needs Inventory, *EuroQol-5D* European Quality of Life questionnaire-5 dimensions, *WHOQOL* World Health Organization Quality of Life questionnaire. *Baseline consists of five assessments and BPDSI, GAF and SOFAS are assessed twice at baseline.

At baseline, the Interview for Traumatic Events in Childhood (ITEC) will be conducted. This is a retrospective, semi-structured interview for childhood maltreatment including sexual, physical and emotional abuse and physical and emotional neglect [28]. At follow-up, patients are assessed for recovery from BPD as well as the most common comorbidities by trained interviewers using the following sections of SCID-I for DSM-IV-Tr: affective disorders (including bipolar disorder I and II), anxiety disorders, eating disorders and substance disorders; and the following sections of SCID-II for DSM-IV-Tr: avoidant, dependent, obsessive-compulsive, paranoid, schizotypal, schizoid, histrionic, narcissistic, antisocial and borderline PD. Since only current diagnoses need to be considered for the assessment of recovery from BPD and comorbidity, the recall period for these shortened SCIDs is limited to six months. Only when a patient will otherwise become a study dropout (e.g. due to unwillingness to come to the centre for assessments), they can fill in questionnaires at home. Interviews are then conducted by telephone. By doing so, the occurrence of missing data will be reduced.

Due to the nature of the study, blinding of participants is not possible. Except for the cost interview, which contains specific questions on which treatments patients have received, assessments will be performed by blinded local research assistants. The cost-interview, containing questions on health care utilization that would unblind the condition will be done by a nonblinded research assistant. This assistant will also monitor treatments provided in TAU. Furthermore, this nonblinded research assistant will collect treatment session recordings that are needed for supervision and validation of treatment adherence. After checking the quality of the recording, the sample of recordings that is needed will be stored.

To maximize adherence to the study protocol, a manual has been created for all local research assistants. A central research assistant is appointed to whom local research assistants can address questions concerning any logistical issues. This individual will also perform checks and provides guidance and direction when needed. Checks include study protocol adherence, in- and exclusion criteria of candidate participants, and keeping track of the scheduling and advancement of assessments, data and the collection of audio and video recordings. The central research assistant will furthermore train local research assistants, distribute the instruments used for assessments, provide updates of the manual, explain data format requirements, and prepare the online data collection environment. In sum, the central research assistant will safeguard the validity of assessments.

### Treatments

#### Group schema therapy

Schemas refer to the knowledge representations people have about themselves, others and the world and which are formed during childhood by the way basic needs are met. When a child has to cope with his or her basic needs not being met, a variety of dysfunctional schemas, and/or coping styles, may develop. Schema modes refer to the emotional states between which BPD patients can rapidly switch when triggered by events that are related to the unmet needs during childhood. Schema modes represent sets of schemas and/or coping styles that are typically expressed in such emotional states. The following schema modes are characteristic for BPD: the vulnerable (abandoned/abused) child, angry/impulsive child, punitive parent, detached protector (or any other protective mode), healthy adult and happy or contented child. The first four of these modes are maladaptive and strongly present in BPD patients. The latter two are functional modes and weak in BPD patients. Schema therapy aims to reduce maladaptive modes and develop and strengthen functional modes [29]. This schema mode model guides therapy as each mode requires a different treatment strategy. The strategies comprise specific experiential, cognitive and behavioural techniques [30]. When offered in a group format, several factors may amplify and speed up recovery: peer support, a sense of belonging and understanding, opportunities for vicarious learning and real-life practice of healthy behaviour [19].

In format A (GST-A), two-year GST consists of 124 groups sessions with a duration of 90 minutes. In the first year group sessions take place twice a week and in the second year the frequency gradually decreases. In addition, in GST-A a total of up to 18 individual sessions can be used at the patients discretion or in times of crisis. Two individual sessions take place before group sessions commence. At this time patients get acquainted with their group schema therapists, schema therapy and the schema mode model are explained, the schema modes a patient has and their relationship to one another are identified and a treatment plan is drawn up.

In format B (GST-B), two-year GST involves a combination of group and individual sessions. In the first year, there are weekly group sessions of 90 minutes and individual sessions of 50 minutes and in the second year the frequency gradually decreases. In total, patients in this condition receive 74 group sessions and 62 individual sessions. In the first two individual sessions patients get acquainted with their individual and group therapists, schema therapy and the schema mode model are explained, the schema modes a patient has and their relationship to one another are identified and a treatment plan is drawn up. Group and individual ST therapists meet regularly to coordinate treatments.

#### Treatment as usual

Following usual procedures, the intake staff at each centre determine the optimal treatment offered to each patient in the TAU condition. Except for (G)ST, the intake staff are allowed a choice from the whole array of possible treatments for BPD with no restriction; whether intensive, individual, group, inpatient, outpatient or day treatment. The TAU condition is thus representative of optimal current practice and will be carefully monitored for all patients. Since there is no protocol for TAU in this RCT and specific treatment is decided on by experts in the community treatment centre, TAU is equivalent to ‘community treatment by experts’.

In some centres, the treatment that is usually offered to BPD patients is Dialectical Behavioural Therapy (DBT). This is an empirically validated treatment for BPD [24,31]. If the number of centres that offer DBT as the usual treatment is sufficient, this will provide an opportunity to compare the clinical effectiveness and cost-effectiveness of GST with DBT.

### Therapists, training and supervision

GST sessions are run by two schema therapists, of whom at least one is a senior schema-therapist. Stand-in schema therapists replace the regular therapists when they are absent (e.g. due to illness, holidays or pregnancy). The senior therapist’s role, in addition to being a group schema therapist, is to act as a local supervisor for other schema therapists. Individual schema therapists can also be group schema therapists for GST. Group schema therapists receive a training of six days in GST [32]. Candidate ST therapists (individual and group) who are not yet trained in ST first receive a 3–4 day training in individual ST for BPD [33]. During the study intensive supervision meetings are held twice a year in the first year and once or twice a year in the second year. In addition, weekly team supervision is provided locally and central supervision by the developers of GST is provided through teleconferencing and viewing of video-recordings weekly in the first months, then biweekly and monthly after about 6 months. Initially, a computer program was acquired that was especially designed to enable secure online sharing of video-recordings of medical procedures through encrypted streaming. Unfortunately, this program has become unavailable. The encrypted recordings will now be uploaded on a central server so that supervisors can download and watch them.

## Evaluation of clinical effectiveness

In this section, the primary and secondary clinical outcome measures that will be used to evaluate the clinical effectiveness of the treatments are described as well as the strategy used for analysis of the data. All instruments that will be used to investigate clinical effectiveness that were not yet available in the languages of all participating sites were translated. Translated versions were back-translated and thoroughly checked for consistency with the original version. Any inconsistencies were addressed in consensus meetings and adjusted accordingly. Instruments are implemented in an online data collection environment.

### Clinical outcome measures

#### Borderline Personality Disorder Severity Index version IV (BPDSI-IV)

The primary outcome measure is the severity of BPD, expressed as a score between 0 and 90 as measured with the Borderline Personality Disorder Severity Index (BPDSI), version IV. The BPDSI-IV is a semi-structured interview containing 70 items based on the nine BPD dimensions described in DSM-IV. This is a reliable and valid instrument, suitable for use as an outcome measure [21,34]. A cut-off score of 15 between patients and controls has been previously established [21,34]. Therefore, a score below 15 measured two years after randomization or earlier and maintained until follow-up can be used as a criterion for recovery. The scores on subscales of the BPDSI-IV provide information on the severity of each of the nine dimensions of BPD. The recall period for the BPDSI-IV is three months.

#### BPD checklist

The BPD checklist is a self-report instrument that measures the burden of BPD manifestations as experienced by patients. Since the BPD checklist measures changes in subjective burden, it is complementary to the BPDSI-IV that measures changes in symptomatology objectively. It consists of 47 items based on the nine dimensions of BPD in DSM-IV and answers are scored on a five point Likert scale. Suitability for use as a treatment outcome measure has been established [35]. The recall period for the BPD checklist is one month.

#### Brief Symptom Inventory (BSI)

The BSI is a self-report instrument used as an inventory of general psychiatric symptoms present at the time of assessment and is a short alternative to the SCL-90-R, from which it was developed [36]. It contains 53 items to inventory the following nine types of primary symptom dimensions: somatic, cognitive, inter-personal sensitivity, depressive mood, anxiety, hostility, phobia, paranoia and psychoticism. Answers are scored on a 5-point Likert Scale. Cronbach’s α is .96 for the instrument in total and ranges between .71 and .87 for its subscales [37]. According to [38], Cronbach’s α values of 0.9 or higher indicate an internal consistency that is appropriate for clinical applications, and values of 0.7 to 0.8 are satisfactory for comparing groups. In addition, the BSI has good discriminant validity [37].

#### Happiness item

The happiness item is a single question on general happiness in the months prior to the assessment and is scored on a seven point Likert scale [39]. This scale consists of the following verbal descriptions of different states of happiness: (1) completely unhappy, (2) very unhappy, (3) fairly unhappy, (4) neither happy nor unhappy, (5) fairly happy, (6) very happy, (7) completely happy. Norms for all participating countries are available [39]. For a single happiness item high test-retest reliability (r = 0.86) and good concurrent, convergent, and divergent validity have been reported [40]. The happiness item has excellent sensitivity to change for patients with BPD who were treated with GST [20].

#### Schema Mode Inventory (SMI)

The SMI is a self-report instrument that consists of 143 items on 16 schema modes that are scored on a six point Likert scale. It measures the extent to which dysfunctional as well as functional schema modes are present at the time of assessment [41]. It is an adaptation of the original SMI containing 270 items [42] and short SMI containing 118 items [43]. This instrument is only used for patients in the GST condition. Its subscales have satisfactory to high internal consistency (Cronbach’s α ranges from .79 to .96) [43]. The SMI is a useful instrument for assessing modes in ST [43].

#### Young Schema Questionnaire – short form (YSQ)

The YSQ is a self-report instrument containing 75 items that are scored on a six point Likert scale [44]. It is used to measure the presence or absence of 16 core maladaptive schemas at the time of assessment. The YSQ has adequate temporal as well as rank-order stability and an analysis of its discriminant power in clinical versus non-clinical samples revealed it is highly sensitive in predicting the presence or absence of psychopathology [45]. Internal consistency is high for the overall scale (Cronbach’s α ranges from .94 to .96) and satisfactory to high for its subscales (Cronbach’s α ranges from .72 to .94) [46].

#### Global Assessment of Functioning (GAF) and Social and Occupational Functioning Assessment Scale (SOFAS)

Based on axis V of DSM-IV, the GAF and SOFAS are 100-point scales used to assess general and social/occupational functioning, respectively. A short semi-structured interview serves to elicit the information needed for scoring. The recall period for both instruments is one month. The GAF is a valid scale of global psychopathology and the SOFAS is a valid measure of social, occupational and interpersonal functioning [47]. Both instruments have excellent interrater reliability (intraclass correlation coefficients > .74) [47].

#### Work and Social Adjustment Scale (WSAS)

The WSAS is a self-report instrument that consists of 5 items that are scored on a scale ranging from 0 to 8. It is used to assess functional impairment at the time of assessment in the domains of work, household, social leisure, private leisure and family and relationships. The WSAS’ reliability, validity and sensitivity to change have been firmly established in samples of patients with different clinical disorders [48-50].

#### Relationship Scales Questionnaire (RSQ)

The RSQ was designed as a continuous measure of adult attachment and consists of 30 short statements about romantic relationships [51,52]. After being instructed to think about such relationships in their past and present lives patients rate the extent that these statements resemble their own feelings and experiences at the time of assessment on a five point Likert scale. Scores are calculated for the following attachment patterns: secure, dismissive, fearful and preoccupied.

#### Emotional Core Needs Inventory (ECNI)

The ECNI is a list of 88 statements used to measure the extent to which basic needs are being met in important relationships at the time of assessment [53]. Each statement is rated on a scale ranging from 1 to 6. A higher rating corresponds to better need-fulfilment. Assessing the extent to which patient’s needs are met by others is important given that maladaptive schemas are hypothesised to result from unmet core emotional needs. A central aim of ST is to bring about changes that lead to better need-fulfilment of patients [54].

#### World Health Organization Quality of Life questionnaire (WHOQOL-short)

The WHOQOL-short is a self-report instrument for assessing quality of life in the two weeks prior to assessment. It is a short version (35 items) of the WHOQOL and focuses on the domains of physical health, psychological health, social relationships, environment, positive feelings, negative feelings and self-esteem. The WHOQOL-short is a reliable and valid instrument [55].

### Analyses

All available data on clinical outcomes will be analysed according to the intention-to-treat principle. Outcome measures will be analysed with mixed regression, also known as multilevel or hierarchical regression, with centre as a random effect, allowing centre by treatment interaction, and including patient-level and treatment indicator covariates, as well as time and treatment by time effects. Baseline covariates will be used to adjust for potential differences at baseline and to reduce standard errors. For categorical outcome variables, counts and in case of non-normal residuals, appropriate forms of mixed regression will be chosen (binomial, Poisson, gamma, etc.).

## Economic evaluation

The following section describes how costs and utilities will be measured for the economic evaluation as well as the planned cost-effectiveness and cost-utility analyses. The cost interview has been translated into the languages of all participating sites. The original Dutch version was first translated to English and subsequently this English version was then translated into Greek and German. The translated versions have been back-translated and/or thoroughly checked for consistency with the original version. Any inconsistencies were addressed in consensus meetings and adjusted accordingly. The cost interview will be implemented in an online data collection environment.

### Costs measurement

Costs will be measured from a societal perspective using a retrospective cost interview especially designed for BPD patients. Relevant costs to be identified include healthcare, patient and family costs, and costs outside the health care sector. Healthcare costs include visits to general practitioners, hospitals, psychiatrists and psychologists, crisis centres, use of medication, social work, formal care, and alternative treatments. Patient and family costs include travelling costs, informal care (care provided by family, friends or neighbours of the patient) and out of pocket costs (alcohol, drugs, smoking and self-reported other costs). Costs in other sectors include productivity losses from unpaid work (voluntary work and study) and paid work. The cost interview will be conducted by trained local research assistants, who will ask questions on the consumption of different resources and assess volumes of resource use. When applicable, calculations and descriptive content will be noted. For the cost interview the recall period is 6 months.

### Utility measures

#### EuroQol-5D-3 L (EQ-5D-3 L)

The EQ-5D-3 L is a generic, self-assessment instrument used for measuring health-related quality of life at the time of assessment [56]. It consists of five questions, each related to a specific dimension of health status: mobility, self-care, usual activity, pain/discomfort and anxiety/depression. The EQ-5D has been translated in the languages of all participating sites [57]. The descriptive profiles, generated by the EQ-5D-3 L are valued using social tariffs for the EuroQol to generate utilities, which reflect a population’s preference for a particular health profile.

In base-case analysis, country-specific tariffs will be used for valuation when available. For the Netherlands, Germany and the UK national tariffs are available, whereas for Greece and Australia they are not [57]. For these countries, proxy tariffs will be used as an alternative (e.g. tariffs for Europe and New-Zealand, respectively). Sensitivity analyses will be performed that make use of country-specific tariffs for the valuation of health profiles of all patients.

Utilities will be used to calculate Quality Adjusted Life Years (QALYs) by multiplying change in utility between assessments by the duration of the time period between assessments. Through the use of statistical regression, potential baseline differences in QALYs can be adjusted for [58].

In addition, the EuroQol thermometer will be scored in a range between 0 and 100 to provide a single index measure for a patient’s health status [56].

### Analyses

The economic evaluation will be comprised of both a cost-effectiveness analysis (CEA) and cost-utility analysis (CUA) and will be performed from a societal perspective. All available data on costs and outcomes will be analysed according to the intention-to-treat principle. Data gathered with the cost interview will first be checked for adherence to questionnaire routing, illogical answers, unrealistic cost estimates and outliers. For any problems in the data that cannot be solved through logic, decision rules will be established. A decision rule could apply a specific limit to volumes of costs, e.g. the maximal hours patients spend per day on caring for their children can be limited to eight for patients who report doing this for 24 hours per day. For intermittent missing values at the item level of the cost interview the mean values of previous and subsequent assessments will be imputed to allow calculation of total costs. Missing values on total scores or other outcomes will not be imputed. Unrealistic or extreme values will be investigated per case and corrected when appropriate. Volumes of resource use as measured by the cost interview will be multiplied by their corresponding unit costs and then summed to provide an overall total cost. Unit costs will be based on standard unit prices for each country (e.g. in the Netherlands: Hakkaart-van Roijen et al. [59] for cost prices of healthcare services [59]) when available and on (averaged) tariffs otherwise. Cost prices will be expressed in Euros for the same base year and indexed using consumer price indices when required. Cost prices expressed in currencies other than Euros will be converted using purchasing power parities. In addition, to account for the three year time horizon of this RCT, cost prices will be discounted according to the guidelines. When neither standard unit prices nor tariffs are available for specific resource items in specific countries, alternative strategies for the valuation of resource use will be considered. When for a subset of resource items the unit costs are known in all countries and for all other resource items there is a unit cost available in at least one country, the unit cost of a resource item that is not available in one or more countries can be estimated through a procedure called market-basket based imputation [60]. In base-case analysis, country-specific unit costs will be used to value resource use for each country. Sensitivity of the results to differences in unit prices between countries will be analysed by valuing resource use in all countries using unit prices of a single country. Productivity losses will be valued by the human capital approach through multiplication of the total number of hours lost with the national average hour wage. Shadow prices will be used to value informal care and lost productivity in study and voluntary work. The primary clinical outcome for the CEA will be the BPD Severity Index (BPDSI) score and for the CUA primary utility scores will be derived from the EuroQol-5D. Cost-effectiveness and cost-utility data will be analysed with multilevel modelling techniques. The net monetary benefit will be used to express cost-effectiveness, and results will be expressed in cost-effectiveness acceptability curves. Net monetary benefit (NMB) will be calculated for a range of values for the amount decision makers are willing to pay (WTP) for an additional unit of effect, following: NMB = ∆E*WTP- ∆C, where ∆E is the difference in effects and ∆C is the difference in costs [61]. To accommodate the skewness that is typically observed in cost data, costs (or NMB) can be assumed to follow a gamma or log-normal distribution. Baseline covariates will be used to adjust for potential differences at baseline and to reduce standard errors. Results will be expressed in cost-effectiveness acceptability curves that display the probability, based on the available evidence, that GST can be considered cost-effective for a range of WTP values. For these types of analyses, a Bayesian approach is the most natural, since it allows direct and intuitive statements to be made regarding the probability that a treatment is cost-effective, based on the available evidence [62]. Furthermore, Bayesian methods allow flexible joint modelling of costs and effects, thereby facilitating sensitivity analyses regarding different methodological approaches for specification and parameterization of the model. Sensitivity analyses will be performed to address uncertainty regarding methodology, model specification and prior distributions.

## Additional substudies

Complementary to effectiveness and cost-effectiveness evaluations, a series of additional investigations will be performed that consist of an assessment of the integrity of GST, an investigation into the opinions of primary stakeholders and additional studies that examine variables that might mediate the change process in GST.

### Treatment integrity

Adherence to GST treatment protocol will be assessed by trained independent judges who rate a random selection of video-recordings of group-ST using a newly developed instrument. For individual ST, a random selection of video-recordings will be rated using existing instruments [16,63] by trained raters. As sampling recordings of TAU will be impossible in many sites, due to ethical and logistic reasons (e.g. TAU in private practice or TAU in mixed groups including patients not participating), the differentiation between ST and TAU will be assessed by having patients fill out a checklist with techniques that are typical for ST and non-typical for ST.

### Patient and therapist perspectives

This study involves the acquisition of qualitative data in the form of patients’ and therapists’ opinions about treatment and the preferred format for GST. The opinions of these major stakeholders will be elicited through in-depth interviews and/or focus groups, allowing them to share their view on treatment and the preferred format for delivery of GST. Topics include which aspects of the GST protocol are perceived to be beneficial or not, application of specific ST techniques, therapeutic relationships and therapist training and supervision. Saturation is expected to be reached after having interviewed 12 GST patients in each participating country and 12–15 therapists, after which assessment will be discontinued. The centres that participate in this aspect of the study are from the Netherlands, Germany and Australia. Centres in other countries might decide to participate later. Patients will be sampled from both GST formats and from 3 phases of treatment: first year, second year, and after treatment completion. All interviews are recorded. After a full transcription of the recordings is made a summary is made. This summary is then reviewed by patients and therapists as a check on its veracity. If needed, the summary will be corrected to be sure that the verbatim transcripts express patient and therapist opinions correctly. Verbatim transcripts of interviews with patients and therapists will be analysed for their content using specialized software. Important and/or recurring themes will be categorized, interpreted and reported.

## Studies on variables affecting treatment outcomes

The final area for additional investigation is the extent that patient variables affect outcomes such as dropout rates and patient improvement. This includes genetic polymorphisms, dissociation, comorbidity, individual patient trajectories, change processes and the therapeutic relationship, changes in neural correlates of emotional sensitivity, regulation and impulsivity during treatment, changes in threat bias during treatment, changes in needs during treatment, somatic symptoms and somatization, the therapist’s voice and use of recordings for secure attachment, the effects of training, early self-understanding as a predictor for outcome, the effect of treatment on comorbidity and changes in attachment representation.

## Discussion

In this article the design is described for an international, multicentre RCT on GST that includes an evaluation of the clinical effectiveness, a full economic evaluation as well as a series of additional investigations. In this RCT, GST (format A and B) will be compared against optimal TAU in terms of clinical effectiveness and cost-effectiveness. Such a design follows the ‘gold standard’ in cost-effectiveness research [64] and allows us to investigate whether GST excels current practice, which consists of the existing optimal treatments that are usually provided to patients with BPD. TAU consists of a variety of different treatments due to the fact clinical practice varies between centres as well as between countries. Since TAU is tailored to the individual needs of each patient, it can be considered representative of optimal current practice. The multicentre and international design of this RCT specifically intends to capture the variation in clinical practice between participating centres and between countries, respectively. Because the resulting amalgam of treatments in TAU reflects current practice, external validity is increased. If the RCT was designed to include a fixed treatment instead of variable TAU as a comparison to GST, then it would be less informative in regard to whether GST excels current practice and whether its further implementation is supported. Furthermore, if the RCT was designed as a head-to-head comparison between GST and another experimental treatment without the inclusion of TAU, then interpretation of its results could be hindered. This is because it is not clear how experimental treatments compare to TAU. For instance, experimental treatments might do worse than or be equivalent to TAU. For these reasons, TAU is considered to be an appropriate comparator to GST at this stage of research. However, with this TAU there is little control over the specific issues that are addressed in therapy, the amount of attention a patient receives and the frequency of therapeutic contacts. It is therefore less rigorously defined than the experimental condition. In addition, therapists providing TAU may not receive the intensive supervision that GST therapists receive and the treatment fidelity of the components of TAU is not monitored. These issues could be a potential threat to internal validity [65]. Notwithstanding, TAU will be delivered by skilful therapists with extensive experience in the treatment of BPD and its contents are monitored by administering a questionnaire on the specific treatments that each patient receives.

The fact that this RCT will take place with multiple participating centres and in an international context has specific advantages and disadvantages. Several advantages of international clinical trials over single-country trials have been formulated [66], which also hold in the case of multicentre trials versus single centre trials. First, in multicentre or international RCTs, through parallel recruitment of patients at the different sites, it takes less time to include a sufficient number of patients in comparison to single site studies. Second, the representativeness of the study population is enhanced by capturing more of the variability in patient characteristics, clinical practice patterns and/or health care systems. Third, the collection of data at multiple sites enables the researcher to inform decision makers in all of the participating sites.

Paradoxically, whereas the inclusion of more variability enhances the representativeness of the study population, this same variability makes it difficult to apply the results to any one centre or country in particular. In other words, studies designed to include multiple participating centres and/or countries raise issues concerning their generalizability [67,68]. At the patient level, variation between sites exists in terms of demography and epidemiology. At the level of treatment centres and clinicians, differences may exist in patient management. Differences between healthcare systems and other socioeconomic factors may influence healthcare delivery and the allocation of scarce resources to healthcare, respectively. Inversely, some degree of similarity within healthcare systems, treatment centres or patients may also be expected. A method that can accommodate the hierarchical structure of such data is multilevel modelling, which has been proposed as an appropriate analytic strategy for cost-effectiveness data from multinational RCTs [67-70]. It allows variation to be estimated within and between the different levels. Moreover, these estimates can be used to calculate centre-specific estimates of cost-effectiveness [67], which can be used to determine the extent to which the results from this RCT are generalizable.

Since this RCT involves multiple participating centres in different countries, organizational and logistical challenges potentially threaten its quality. Handling these challenges is a labour-intensive process that requires thoughtful planning, a clear protocol, continuous monitoring of protocol adherence, and well-defined communicational lines. The hub in the logistical infrastructure is a central research assistant who will perform checks and steering concerning study protocol adherence and therefore plays an important role in ensuring the validity of the assessments. Organizational issues may arise when, for example, therapists, coordinators or research assistants retreat from the study and are replaced, when recruitment rates are slower than was foreseen or video-facilities for treatment supervision are missing. The appropriate handling of these issues requires timely noticing of their occurrence, which will be facilitated by regular internet conferences involving the principal investigators and local coordinators.

In this RCT, both primary and several secondary clinical outcome measures will be assessed through interviews. Therefore, it is necessary to control interviewer bias by having blinded interviewers perform the assessments. For interviews containing specific questions about which treatment the patient receives (the cost interview and the monitoring of treatments provided in TAU), blinding of interviewers will obviously not be possible. These interviews will be performed by non-blinded research assistants. All other interviews and questionnaires will be performed and administered by blinded research assistants.

When conducting an economic evaluation in the field of mental health, it can be a challenge to provide a comprehensive account of all the costs and consequences that are associated with the treatments being compared and which are relevant under the target perspective [71]. Since relevant costs for BPD include health care costs, patient and family costs and costs in other sectors, a societal perspective will be taken. This also prevents cost shifting to be interpreted as increases or decreases in costs. The time horizon of three years covers both the duration of the GST treatment as well as a one year follow-up time period. This enables an investigation into the stability of treatment outcome over time. In addition, relevant costs that are incurred once the GST treatment has ended, which could be a consequence of treatment outcome, are thus included. To gather data on the societal costs and consequences that are associated with BPD and the BPD treatments being compared in this RCT, resource use in a wide range of health care facilities is taken into account, whether inpatient or outpatient, including various health services specialized for mental health (e.g. contacts with a psychologist or psychiatrist) as well as more general health care services (e.g. contacts with a GP or general hospital). Furthermore, costs due to productivity losses and informal care will be measured to take into account the fact that costs and consequences have an impact on society as well as family and friends, respectively. Lastly, by taking into account various categories of out-of-pocket expenses that are typically associated with BPD (e.g. alcohol, tobacco and drug use, impulsive buying, binge eating) an attempt will be made to measure all relevant costs that are specifically associated with BPD.

Despite the extensive effort that is put into obtaining a complete picture of the costs and consequences that are associated with BPD and the BPD treatments being compared in this RCT, it remains unfeasible to include some particular aspects. For instance, to date no instruments exist to measure the high burden that BPD patients can impose upon colleagues and organizations due to suboptimal functioning at work [72]. Also, leisure time is relevant to patients with BPD, but it can be difficult to measure and value. Therefore, these aspects are not taken into account as costs or consequences in the economic evaluation.

Another degree of complexity is added to economic evaluations in the field of mental health, as opposed to somatic disorders, due to the fact that once costs and consequences are measured, their interpretation can be difficult. No consensus exists on the extent to which particular costs need to be included or not and how they need to be valued [73]. For example, although informal care provided by family and friends is very relevant to patients with BPD, it can be difficult to know which amount of care is specifically due to mental health problems and which amount they would have received anyway. Similarly, productivity losses can be the result of being under treatment, yet patients with mental health problems are also less likely to be employed or could have already lost their job before having their diagnosis [74]. Furthermore, although the volumes of production losses in paid work, voluntary work, study, and household activities as well as contacts with the GP and medication use are explicitly measured separately for being BPD-related or not, this division cannot always be reliably made. For example, a leg injury by itself may seem unrelated to BPD at first sight, but less so when it is the result of a suicide attempt. In such cases, the analyst is guided by the information provided, while at the same time being aware that this information may or may not be complete. In cases where there is sufficient information to attribute a somatic complaint to an underlying mental health complaint, patient answers are overruled. Reliability can also be an issue when rather high out-of-pocket expenses are reported [72].

In addition to the evaluation of clinical effectiveness and the economic evaluation, a series of additional investigations will be performed in this RCT that consist of an assessment of the integrity of GST, in terms of adherence to the GST protocol, an investigation into the opinions of major stakeholders and analyses of variables that might mediate treatment response. The qualitative data on the experiences of patients and therapists are considered as complementary to the quantitative methods that will be employed in this study. By interviewing patients and therapists, potentially important, yet unanticipated, issues may be detected. Furthermore, this type of data collection can give valuable insight into the contextual factors that play a role in the effectiveness of GST and its implementation.

## Conclusion

GST holds much promise as a treatment for BPD. However, since only two small studies have tested GST important questions remain to be answered before its further implementation is supported. The current international, multicentre RCT is designed to reveal how GST, when delivered as a complete and stand-alone treatment by therapists who were not involved in its development, compares to up-to-date TAU. Concurrently, this RCT aims to investigate the optimal format for the delivery of GST; whether consisting almost exclusively of group sessions or as a combination of individual and group sessions.

In addition to an investigation of clinical effectiveness, this international multicentre RCT will involve an economic evaluation to investigate how GST compares to TAU, and how both formats for GST compare in both clinical and economic terms. Furthermore, a series of additional investigations will be performed to shed light on the qualitative aspects of GST and on variables that influence treatment outcome. In sum, this RCT contributes to an evidence-based understanding that will inform decisions regarding which treatment to offer to patients with BPD, both from a clinical and societal perspective.

## Additional file

Additional file 1:
**Sample size calculation for multicentre RCT on GST for BPD.**
