# Correction to: Design of an international multicentre RCT on group schema therapy for borderline personality disorder

**DOI:** 10.1186/s12888-022-03825-2

**Published:** 2022-03-25

**Authors:** Pim Wetzelaer, Joan Farrell, Silvia M. A. A. Evers, Gitta A. Jacob, Christopher W. Lee, Odette Brand, Gerard van Breukelen, Eva Fassbinder, Heather Fretwell, R. Patrick Harper, Anna Lavender, George Lockwood, Ioannis A. Malogiannis, Ulrich Schweiger, Helen Startup, Teresa Stevenson, Gerhard Zarbock, Arnoud Arntz

**Affiliations:** 1 Department of Clinical Psychological Science, Faculty of Psychology and Neuroscienc, Maastricht University, Universiteitssingel 40, 6229 ER Maastricht, P.O. Box 616, 6200 MD Maastricht, The Netherlands; 2 Department of Psychology, Indiana University-Purdue University Indianapolis, Administrative Office, 402 N Blackford, LD 124, Indianapolis, IN 46202 USA; 3 Center for Borderline Personality Disorder Treatment & Research, Indianapolis, USA; 4 Department of Health Services Research, CAPHRI School of Public Health and Primary Care, Faculty of Health Medicine and Life Sciences, Maastricht University, Duboisdomein 30, 6229 GT Maastricht, P.O. Box 616, 6200 MD Maastricht, The Netherlands; 5 Trimbos Institute, The Netherlands Institute of Mental Health and Addiction, Utrecht, The Netherlands; 6 Department of Clinical Psychology and Psychotherapy, Institute for Psychology, University of Freiburg, Engelbergerstrasse 41, 79085 Freiburg, Germany; 7 Department of Psychology and Exercise Science, Murdoch University, 90 South St, Murdoch, WA 6153 Australia; 8 De Viersprong, The Netherlands Institute for Personality Disorders, De Beeklaan 2, Postbus 7, 4661 EP Halsteren, The Netherlands; 9 Department of Methodology and Statistics, Faculty of Health Medicine and Life Sciences, Maastricht University, Peter Debyeplein 1, P.O. Box 616, 6200 MD Maastricht, The Netherlands; 10 Faculty of Psychology and Neuroscience, Maastricht University, Universiteitssingel 40, P.O. Box 616, 6200 MD Maastricht, The Netherlands; 11 Department of Psychiatry and Psychotherapy, University of Lübeck, Ratzeburger Allee 160, 23538 Lübeck, Germany; 12 Midtown Mental Health/ Eskenazi Health, 5610 Crawfordsville Rd Suite 22, Indianapolis, IN 46224 USA; 13 Department of Psychiatry, Indiana University School of Medicine, Indianapolis, USA; 14 Bradford District Care Trust, Bradford, UK; 15 South London and Maudsley NHS Foundation Trust, London, UK; 16 Schema Therapy Institute Midwest, 471 West South Street, Suite 41C, Kalamazoo, MI 49007 USA; 17 1st Department of Psychiatry, Eginition Hospital, Medical School, Athens University, 72-74, Vas. Sofias Ave, 115 28 Athens, Greece; 18 Greek Society of Schema Therapy, 17, Sisini str, 115 28 Athens, Greece; 19 Klinik für Psychiatrie und Psychotherapie, University of Lübeck, Ratzeburger Allee 160, 23538 Lübeck, Germany; 20 Peel and Rockingham Kwinana Mental Health Service, Cnr Clifton and Ameer Street, P.O. Box 288, Rockingham, WA 6968 Australia; 21 IVAH GmbH (Institute for Training in CBT), Hans-Henny-Jahnn-Weg 51, 22085 Hamburg, Germany; 22 Department of Clinical Psychology, University of Amsterdam, Weesperplein 4, 1018 XA Amsterdam, The Netherlands

**Correction to: BMC Psychiatry 14, 319 (2014)**

**https://doi.org/10.1186/s12888-014-0319-3**

Following publication of the original article [1], the authors identified errors in the numbers of the below sentences. The updated numbers are given below and the changes have been highlighted in **bold typeface**.

The sentences currently read:

In format A (GST-A), two-year GST consists of 124 groups sessions with a duration of 90 minutes.

In addition, in GST-A a total of up to 18 individual sessions can be used at the patients discretion or in times of crisis.

In total, patients in this condition receive 74 group sessions and 62 individual sessions.

The sentences should read:

In format A (GST-A), two-year GST consists of **118** groups sessions with a duration of 90 minutes.

In addition, in GST-A a total of up to **17** individual sessions can be used at the patients discretion or in times of crisis.

In total, patients in this condition receive **63** group sessions and **61** individual sessions.

The original article [1] has been corrected.
