# Supplementary material for: Design of an international multicentre RCT on group schema therapy for borderline personality disorder
Source: BMC Psychiatry. 2014 Nov 18;14:319. doi: 10.1186/s12888-014-0319-3 (PMC4240856; doi:10.1186/s12888-014-0319-3)
Supplement: Additional file 1: — Sample size calculation for multicentre RCT on GST for BPD. [file 12888_2014_319_MOESM1_ESM.pdf]

APPENDIX A Sample size calculation multicentre trial of group-ST for BPD

Design:
Multicentre trial in 5 countries, 14 centres, a minimum of 2 cohorts of 16 patients per centre, randomization to group schema therapy (GST) versus treatment as usual (TAU) per cohort per centre. There are two formats for GST, GST-A and GST-B. Per centre one cohort will get GST-A or TAU, and the other cohort will get GST-B or TAU. Order of treatment will be balanced between centres; in half of the centres, cohort 1 will get GST-A or TAU and cohort 2 will get GST-B or TAU, and in the other half of the centres cohort 1 will get GST-B or TAU and cohort 2 will get GST-A or TAU.

Hypotheses:

1) The outcome mean under group schema therapy (GST-A or GST-B) will be higher than under TAU.
2) The outcome mean under GST-A will differ from the outcome mean under GST-B.

Analysis:
Data will be analyzed with mixed (multilevel) regression to take nesting of patients within centres into account, and adjusting for country, cohort and the difference between treatment A and B. For quantitative outcomes, the following mixed linear model will be applied for the outcome of patient *i* in centre *j*:

$$Y_{ij} \quad = \quad b_{0j} + b_{1j}\, cohort_{ij} + b_{2j}\, treatA_{ij} + b_{3j}\, treatB_{ij} + e_{ij} \qquad (1)$$

where cohort, treatA and treatB are dummy indicator variables for cohort (1 = cohort 2, 0 = cohort 1), GST-A (1 = GST-A, 0 = GST-B or TAU) and GST-B (1= GST-B, 0 = GST-A or TAU), respectively.
The regression weights are the sum of a fixed average weight *β* and a random centre effect *u*, thereby allowing for a centre main effect, centre by cohort interaction, and centre by treatment interaction, respectively. The four random effects are allowed to covary. The last term, e(ij), is the residual representing a random patient effect plus measurement error. The fixed model part will be extended with country effects using dummy coding, with cohort by treatment interaction, and with relevant covariates to increase power and to test for any hypothesized treatment by covariate interactions.

The model assumes one outcome measurement per patient. Repeated measures will be aggregated into a powerful summary measure following the methods in Frison and Pocock (1997) and Senn, Stevens & Chaturvedi (2000). In case of a substantial percentage of missing values, this method will be replaced with three-level mixed regression analysis, adding time of measurement as third level below the patient level, and choosing as model for the treatment by time interaction the same model that underlies the choice of summary measure, i.e. linear divergence between treatment arms over time, allowing for nonlinear trend within each arm.

The model treats centre as a random effect. If the number of centres is too small for stable estimation of centre effects after adjusting for country, then centre will be included as fixed effect. This gives a smaller sample size than the present calculation, at the price of restricting all inferences to the centres in this trial.

Sample size calculation for a quantitative outcome and hypothesis 1:

Since country, centre and cohort are orthogonal to both treatment indicators due to the design chosen, their fixed and random effects can be ignored in treatment effect estimation, giving the following contrasts of interest for hypothesis 1:

$$[(\mu_A + \mu_B)/2 \quad - \quad (\mu_{TA} + \mu_{TB})/2] \quad \neq \quad 0$$

where μ_A and μ_B are the expected outcomes under treatments GST-A and GST-B, and μ_{TA} and μ_{TB} are the expected outcomes under the TAU control to GST-A condition and under the TAU control to GST-B condition respectively. This contrast can be estimated by using the sample means per centre and averaging these across centres, assuming a sample size of n = 8 per treatment condition per cohort per centre.
Using model (1), the variance (= squared standard error) of this contrast estimator can be shown to equal:

$$\left(\sigma_2^2 + \sigma_3^2 + 2\sigma_{23} + \frac{4\sigma_e^2}{n} \right) / \, 4K \qquad (2)$$

where *K* = the number of centres, *n* = the nr of patients per centre per cohort per treatment arm (we assume *n* = 8), and the variances are the between-centre variance of the GST-A effect, the between-centre variance of the GST-B effect, the between-centre covariance of the two effects, and the within-centre between-patient outcome variance. Assuming the between-centre variances of GST-A effects and GST-B effects to be equal, the worst-case scenario is when the two treatment effects correlate perfectly between centres, reducing (2) to $\left(\sigma_2^2 + \frac{\sigma_e^2}{n} \right) / \, K$, which is the same expression as for a multicentre trial with only one experimental and one control treatment arm and a total of 4*n* patients per centre (Moerbeek, Van Breukelen & Berger, 2000, 2003, noting that they used -1/+1 instead of 0/1 treatment coding which makes the treatment effect estimator, and its standard error twice as small, and σ_2^2 four times as small, as presently, for technical details, see Van Breukelen, 2013). Without centre by treatment interaction (i.e. if $\sigma_2^2 = 0$), it follows from standard sample size formulae (e.g. Kirkwood, 1988) that a total of 168 patients (= 5.25 centres) is sufficient to detect an effect size of *d* = 0.50 with 90% power using a two-tailed α of 5%. Assuming centre by treatment interaction such that σ_2^2 is 5% of the between-patient σ_e^2 (which gives a typical intraclass correlation value of almost 0.05), we need 236 patients or 8 centres. Including 13 centres of 32 patients each will then give sufficient power for an effect size *d* = 0.40.

Sample size adaptation to hypothesis 2:

The contrast of interest is now $\mu_A - \mu_B$ and the data from TAU do not add useful information here. In particular, subtracting from $\mu_A - \mu_B$ the term $\mu_{TA} - \mu_{TB}$ to adjust for cohort effects is superfluous as model (1) already adjusts for cohort effects, and will increase the standard error of the effect estimator. Hypothesis 2 is tested by running model (1) without the TAU patients and dropping the treatB indicator such that GST-B is reference treatment against which GST-A is compared. The contrast of interest has variance

$$\left(\sigma_2^2 + \frac{2\sigma_e^2}{n}\right) / K \qquad (3)$$

where σ_2^2 will have a different value than in model (1) for hypothesis 1, as it now reflects between-centre variance in the outcome difference between GST-A and GST-B rather than between GST-A and TAU. Compared with the result for hypothesis 1, the between-patient variance σ_e^2 counts twice since each treatment arm (GST-A versus GST-B) has now *n* patients per centre, against 2*n* for hypothesis 1 (GST-A and GST-B pooled versus TAU). Ignoring intraclass correlation gives a sample size of 168 patients (= 10.5 centres assuming 16 patients on treatment GST-A or GST-B per centre) to detect an effect of medium size *d* = 0.50 with 90% power using a two-tailed α of 5%. Taking again an intraclass correlation of nearly 0.05, we need a sample size of 202 patients (= 13 centres).

In short, a total of 13 centres will give a 90% power to detect an effect of size *d* = 0.40 for hypothesis 1 (TAU versus GST), and an effect of size *d* = 0.50 for hypothesis 2 (GST-A versus GST-B).Taking into account 5% attrition we thus need a total of 14 centres.

References:

Kirkwood, B.R. (1988). Essentials of medical statistics. Oxford (UK): Blackwell.

Frison, L., & Pocock, S. (1997). Linearly divergent treatment effects in clinical trials with repeated measures: Efficient analysis using summary statistics. *Statistics in Medicine, 16,* 2855-2872.

Moerbeek, M, Van Breukelen, G, & Berger, M (2000). Design issues for experiments in multilevel populations. *Journal of Educational & Behavioral Statistics*, *25*, 271-284.

Moerbeek, M, Van Breukelen, G, & Berger, M (2003). A comparison between traditional methods and multilevel regression for the analysis of multi-center intervention studies. *Journal of Clinical Epidemiology, 56,* 341-350.

Senn, S., Stevens, L., & Chaturvedi, N. (2000). Repeated measures in clinical trials: Simple strategies for analysis using summary measures. *Statistics in Medicine, 19,* 861-877.

Van Breukelen, G (2013). Optimal experimental design with nesting of persons in organizations. *Zeitschrift fuer Psychologie, 221(3)*, 145-159.
